# Cenobamate as add-on treatment in ultra-refractory focal epilepsy: Real-world results from The Danish Epilepsy Centre, Dianalund, Denmark

**DOI:** 10.1007/s10072-025-08174-y

**Published:** 2025-04-15

**Authors:** Cathrine E. Gjerulfsen, Stefan Juhl, Katarzyna M. Mieszczanek, Lucie Spanilá, Kristin S. Thygesen, Agnieszka Pavbro, Rikke S. Møller, Guido Rubboli

**Affiliations:** 1 Department of Epilepsy Genetics and Personalized Medicine, Danish Epilepsy Centre, member of the European Reference Network EpiCARE, Visby Allé 3, 4293 Dianalund, Denmark; 2 Department of Regional Health Research, Faculty of Health Sciences, University of Southern, Odense, Denmark; 3 Adults with Epilepsy – Neurological Department, Danish Epilepsy Centre, Dianalund, Denmark; 4 Institute of Clinical Medicine, University of Copenhagen, Copenhagen, Denmark

**Keywords:** Drug-resistant epilepsy, Focal epilepsy, Refractory, Anti-seizure medication, Cenobamate

## Abstract

**Objectives:**

At The Danish Epilepsy Centre, Dianalund, Denmark, we aimed to assess the long-term efficacy, tolerability profile, and influence on concomitant antiseizure medications (ASMs) of cenobamate as an add-on treatment in patients with ultra-refractory focal epilepsies.

**Methods:**

Adult patients with ultra-refractory epilepsy (defined as focal epilepsies in which ≥ 6 treatments, including ASM trials, epilepsy surgery, and vagus nerve stimulator, failed to achieve seizure control) treated with add-on cenobamate between October 2021 and June 2024 were included in our retrospective, observational study. Data were collected through electronic patient records and seizure-diaries.

**Results:**

32 patients were included. The mean length of treatment was 21 months (range 2–32 months) and the median dose of cenobamate was 250 mg (range 50-400 mg). Fourteen/32 (44%) patients were responders (≥ 50% reduction in seizure frequency) to cenobamate, including five patients who became seizure-free (15.6% of the total cohort). Eleven/32 (34%) discontinued due to adverse effects (AEs) or lack of efficacy. Patients with active focal-to-bilateral-tonic–clonic seizures remaining on treatment decreased by 50%. The ASM most frequently reduced was lacosamide, while the one most often discontinued was lamotrigine. Fifteen/32 (47%) patients reported at least one AE during the treatment period of 32 months. Two-thirds were resolved by dose-reduction of ASMs or cenobamate. AEs most frequently reported were tiredness and dizziness; the lowest incidence of these AE was found when cenobamate was added as the third drug.

**Significance:**

Our study underlines the usefulness of cenobamate in treating patients with ultra-refractory epilepsy and indicates its long-term effectiveness in real-world clinical practice.

## Introduction

In the treatment of epilepsy, seizure remission without intolerable or harmful adverse events is the ultimate goal. However, 30–40% of patients with epilepsy do not achieve adequate seizure control despite the availability of a large selection of anti-seizure medications (ASM) and the introduction of several new drugs in the last few years [1]. Drug-refractory epilepsy, defined as the failure of two appropriately selected and tolerated ASM regimes [2], is associated with several unmet needs [3], which negatively impact patients’ employment status and quality of life in general [4]. The term “ultra-refractory” epilepsy has been applied to those epilepsies in which at least six different treatments (well-tolerated ASM trials, epilepsy surgery, vagus nerve stimulator (VNS)) failed to achieve seizure control [5]. Thus, ultra-refractory epilepsy defines a group of severe epilepsies with minimal chances of attaining seizure freedom.

Cenobamate (CNB) is the most recent ASM made available for the add-on treatment of drug-resistant focal epilepsies. It is characterized by a unique dual-action mechanism, that includes the blockade of persistent sodium currents and positively modulates GABA_A_ receptors at a site distinct from the binding site of benzodiazepines [6, 7]. Two randomized, double-blind, placebo-controlled trials have proved the effectiveness of add-on CNB in treating adult patients with drug-resistant epilepsy [8, 9]. Also, CNB has been found safe and to maintain its efficacy in long-term open-label extension studies [10, 11], and a meta-analysis has documented the efficacy and safety of CNB in real-world settings [6]. The positive treatment outcomes of CNB might be related to the long half-life, estimated to be 50–60 h within the therapeutic dosing range [12], which reduces the chance of seizure relapse in case of occasional missing doses. A recent systematic review evaluated CNB to be the highest-ranked third-generation ASM regarding efficacy [13], although its clinical use may be limited by low tolerability at high doses and reports of drug reaction with eosinophilia and systemic symptoms (DRESS) [7, 14].

In this study, we present the result of a real-life single-center observational study in patients suffering from ultra-refractory focal epilepsy treated with add-on CNB, followed at The Danish Epilepsy Centre, Dianalund, Denmark. We aimed to evaluate the long-term efficacy and tolerability of add-on CNB in this severe group of epilepsies, and its impact on concomitant medication in clinical practice during the longest treatment period reported so far in a real-world study with CNB.

## Methods

### Participants and study design

All adult patients (> 18 years) with ultra-refractory focal epilepsy (defined as failure to ≥ 6 epilepsy treatments including ASM trials, epilepsy surgery, and VNS) [5] treated with CNB between October 2021 and June 2024 were included in the study. Patients were treated by epileptologists with an established experience in treating epilepsy. Prior to inclusion, consent was obtained from the patients to review their electronic medical records and publish the results anonymously. Due to the study design, an ethical committee approval was not required.

### Data collection and outcome measures

Through electronic medical records and patient seizure diaries, data on demographics, etiology, seizure types and frequency, CNB dose and treatment duration, adverse effects to treatment, concomitant ASMs, earlier failed ASMs, epilepsy surgery, and treatment with VNS or ketogenic diet were collected. Epilepsy and seizure types were classified according to the latest ILAE classification proposals [15]. Data on seizure frequency recorded in the electronic medical records and seizure diaries in the three months prior to CNB initiation were used to retrospectively estimate the baseline monthly seizure frequency. Patients with at least one focal to bilateral tonic–clonic seizure (FBTCS) in the preceding year were defined as having “active FBTCS”[16]. Responders to treatment were defined as those with ≥ 50% reduction in seizure frequency. The patients were categorized as seizure-free if seizures had not occurred for 12 months or three times the longest pre-intervention inter-seizure interval [2].

The primary outcome measure was the reduction in monthly seizure frequency compared with baseline, while secondary outcomes were changes in the number of concomitant ASMs and number of patients having active FBTCS. Treatment adverse effects, reasons for discontinuation of CNB, and the combination of drugs with the best seizure outcome were also analyzed.

## Results

### Cohort demographics and concomitant treatment

A total of 32 patients with ultra-refractory epilepsy were included. Baseline demographics are summarized in Table 1. One patient did not comply with the criteria for ultra-refractory epilepsy, since she had failed only four trials of epilepsy treatment. However, the patient was included in our cohort due to the severity of her drug-resistant epilepsy with high seizure frequency. In our cohort, 59% of the patients were women, and the mean age was 39 years. The etiology was structural in 11/32 (34%) patients (e.g. mesial temporal sclerosis, bilateral cortical dysplasia, polymicrogyria, perisylvian syndrome, developmental venous abnormalities, and periventricular heterotopia), and infectious in 1/32 (3%). At baseline, 50% of the patients had more than 10 monthly seizures and an additional 12.5% had between 5 and 10 seizures per month, consisting of focal seizures with impaired awareness (FSIA), focal seizures without impaired awareness (FSWIA), or FBTCS. FSIA was the most frequent seizure type, being reported in 27/32 (85%) patients. Additionally, 11/32 (34%) patients had active FBTCS at baseline. Seven/32 (22%) patients had previously undergone epilepsy surgery, 3/32 had a VNS and 1/32 was treated with ketogenic diet. The median number of earlier epilepsy treatments per patient (including well-tolerated ASM trials, epilepsy surgery, and VNS) was 8.5 (range 4–20).

**Table 1:** Baseline characteristics of the 32 patients with ultra-refractory and drug-resistant focal epilepsy treated with cenobamate

Baseline characteristics (*n* = number of patients)	32
Age, mean (range)	39 years (23–72)
Female, *n* (%)	19 (59%)
Epilepsy type, *n* (%)	
Unifocal	21 (66%)
Multifocal	11 (34%)
Etiology, *n* (%)	
Structural	11 (34%)
Infectious	1 (3%)
Unknown	20 (63%)
Intellectual disability, *n* (%)	7 (22%)
Seizure type, *n* (%)	
FSIA	27 (84%)
FSWIA	9 (28%)
FBTCS	9 (28%)
Frequency of seizures at baseline in total, *n* (%)	
≤ 5 per month	12 (37.5%)
< 5 and ≥ 10 per month	4 (12.5%)
> 10 per month	16 (50%)
Concomitant ASMs at baseline	
Average number of ASM	
1 ASM, *n* (%)	2 (6%)
2 ASMs, *n* (%)	14 (44%)
3 ASMs, *n* (%)	7 (22%)
4 ASMs, *n* (%)	7 (22%)
5 ASMs, *n* (%)	2 (6%)
Failed trials of epilepsy treatments before cenobamate initiation, mean (range)	8.5 (4–20)
Prior epilepsy surgery	
Yes, *n* (%)	7 (22%)
Additional anti-seizure therapy	
VNS, *n* (%)	3 (9.7%)
Ketogenic diet, *n* (%)	1 (3.2%)

### Retention rate and cenobamate dose

Each patient was followed from CNB initiation until database closure in June 2024. In all patients, the prescribing guidelines for CNB titration were followed, and the maximum dose given was 400 mg [17]. The dose established as the maintenance dose per patient was the highest-reach dose of cenobamate at the end of the study period. All patients reached the maintenance dose within 8–24 months, and all patients, at the end of the study period, were treated with the maintenance dose for at least 7 months before data collection.

**Fig. 1:**
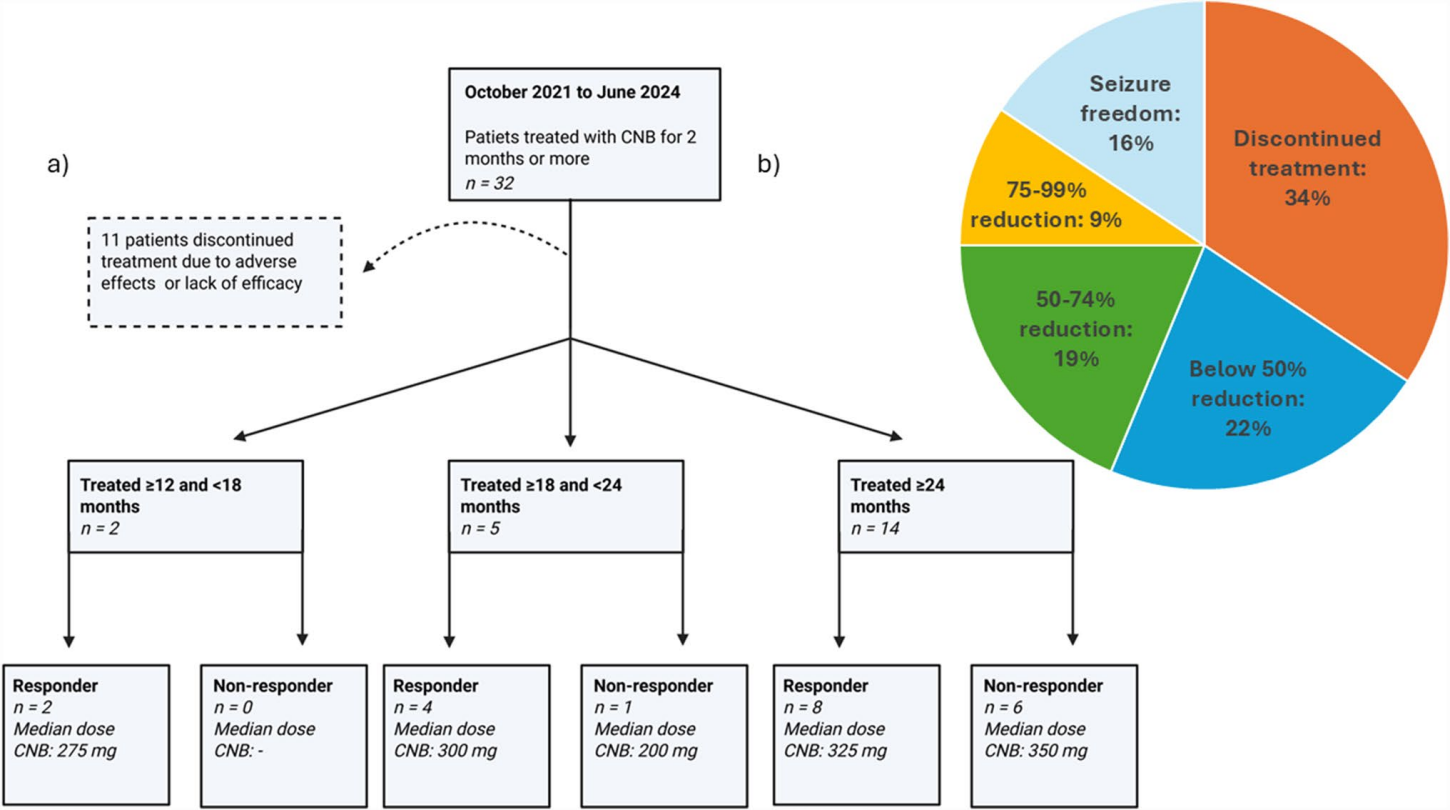
The distribution of responders and the retention of patients treated with cenobamate in the study period lasting from October 2021 to June 2024. The percentage distribution of response to add-on treatment with cenobamate in our cohort of 32 patients is also illustrated

By June 2024, 66% (21/32) of the patients were still treated with CNB (Fig. 1a) of which 2/21 (10%) had been treated for ≥ 12 and < 18 months (median dose 275 mg, range 250–300 mg) by the end of the study period. The remaining 5/21 (24%) patients and 14/21 (67%) had been treated for ≥ 18 and < 24 months (median dose 300 mg, range 200–400 mg) and for ≥ 24 months (median dose 300 mg, range 100–400 mg), respectively. Thus, the total retention rate after at least one year of treatment was 78%. Of the total cohort, the mean length of treatment was 21 months (range 2–32 months), including five patients treated less than three months. The median dose of CNB was 250 mg (range 50–400 mg) (Fig. 1a), of which two patients received doses of CNB of 50 mg daily.

During the study period, 11/32 (34%) patients discontinued CNB; 7/11 discontinued due to adverse effects (tiredness, impaired balance/dizziness, diarrhea, anxiety), and 3/11 discontinued treatment due to lack of efficacy in combination with adverse effects. Additionally, one patient discontinued after 19 months of treatment due to lack of efficacy. The median treatment duration for those who discontinued treatment was 6.5 months (range 2–19 months) and they reached a median dose of CNB at 100 mg (50–250 mg) before discontinuation.

### Efficacy

The efficacy of treatment with CNB was evaluated by dividing patients into groups of responders (≥ 50% reduction in seizure frequency) and non-responders (< 50% reduction in seizure frequency). By the end of the study period, 14/32 (44%) patients were responders, including five patients who became seizure-free (15.6% of the total cohort), three who achieved a 75–99% seizure reduction (9% of the total cohort), and six patients who achieved a 50–74% reduction in seizures (18.8% of the total cohort) (Fig. 1b). Of the five seizure-free patients, one patient did not present seizures for 27 months, another for 19 months, and another one for 15 months. The remaining two patients were without seizures for nine and seven months, which was three times the longest period without seizures since epilepsy onset. The seizure-free patients were treated with a median dose of CNB of 250 mg (range 100–350 mg); the median length of the treatment period was 19 months (range 17–32 months). All five patients suffered from FSIA, and baseline seizure frequency was 3–4 FSIA/month in four of the cases and 2 FSIA/day in the last patient. The 3/14 responders (9% of the total cohort) who achieved a 75–99% seizure reduction, had a baseline frequency of FSIA and FSWIA ranging from 5 to 16 seizures per month, and one patient had active FBTCS, which disappeared when treated with CNB. These patients were treated for a mean period of 22 months (range 18–29 months) with a median maintenance dose of CNB at 350 mg (range 150–400 mg). Additionally, 6/14 responders (18,8% of the total cohort) who achieved a reduction in seizure frequency at 50–74% over a median treatment period of 26 months (range 17–32 months), were treated with a median maintenance dose of 250 mg (range 200–400 mg).

**Fig. 2:**
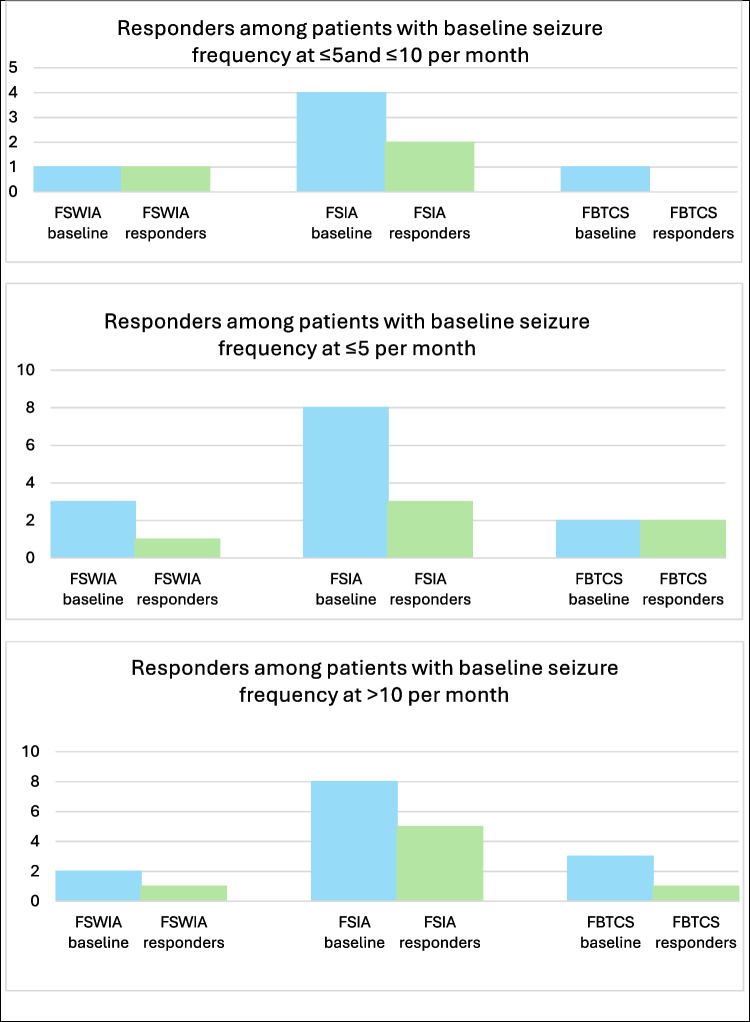
Responders among patients with baseline seizure frequencies at ≤ 5 per month, at ≤ 5 and ≤ 10 per month, and > 10 per month, with seizures divided into different types according to the latest ILAE classification proposals [1]

In the group of 21 patients, who continued CNB, 20 patients suffered from FSIA, which was the predominant seizure type at baseline (Fig. 2). In 50% of the patients suffering from FSIA, add-on treatment with CNB led to a reduction above 50% of this seizure type, and the greatest response was observed in the group of patients with a baseline seizure frequency of ≤ 5 seizures per month. Six patients suffered from FSWIA at baseline, and 50% of those experienced a reduction of FSWIA above 50% after the introduction of CNB. Additionally, 6/21 patients (29%) had active FBTCS at baseline of which 3/6 (50%) were free of FBTCS at the end of the study period.

Of the total cohort, 7/32 (22%) had a seizure reduction below 50% and were therefore defined as non-responders. Compared to the baseline seizure frequency, the non-responders had a mean reduction in seizure frequency of 13.3% (range 0–30%). The median length of the treatment period was 27 months (range 19–31 months), and a median maintenance dose of 325 mg (range 200–400 mg) was reached.

Overall, no correlation between etiology and response to CNB was observed.

### Concomitant ASMs

Information regarding the changes in concomitant ASMs during the treatment period was obtained to evaluate which combinations were the most effective and to estimate if it was most favorable to add CNB to concomitant treatment as the third, fourth, fifth, or sixth drug in our cohort. At baseline, most patients were treated with 2–4 concomitant ASMs (Table 1), of which the most common were lacosamide (17/32), levetiracetam (15/32), clobazam (9/32), and lamotrigine (9/32).

During the study period, 31 adjustments of concomitant treatment consisting of dose reduction or discontinuation were performed in 19/32 patients. The most frequently ASM reduced in dose was lacosamide, and the most frequently discontinued ASM was lamotrigine (Table 2). Other ASMs adjusted in dose or discontinued were carbamazepine, clobazam, levetiracetam, zonisamide, oxcarbazepine, perampanel, brivaracetam, valproic acid, topiramate, and phenytoin. After the initiation of CNB, none of the patients had an additional ASM added or an increment of the dosage of concomitant ASMs.

**Table 2:** Overview of discontinuations and dose-reductions of concomitant ASMs after the introduction of cenobamate

	Concomitant ASMs at baseline, *n* (%)	ASM dose reduction, *n*	ASM discontinuations, *n*
LCS	17/32 (53%)	7	1
LEV	15/32 (47%)	1	1
LMT	9/32 (28%)	2	3
CLB	9/32 (28%)	0	2
CBZ	7/32 (22%)	2	0
ZNS	5/32 (16%)	0	2
BRV	5/32 (16%)	2	1
TPM	5/32 (16%)	1	0
PER	4/32 (13%)	0	2
CNZ	3/32 (9%)	0	0
PHE	2/32 (6%)	1	0
VPA	2/32 (6%)	1	0
OXC	2/32 (6%)	1	0
ESL	1/32 (3%)	1	0
RUF	1/32 (3%)	0	0
CBD	1/32 (3%)	0	0
PGB	1/32 (3%)	0	0

*BRV* Brivaracetam; *CBZ* Carbamazepine; *CBD* Cannabidiol; *CLB* Clobazam; *CNZ* Clonazepam; *ESL* Eslicarbazepine; *LCS* Lacosamide; *LEV* Leveiracetam; *LMT* Lamotrigine; *OXC* Oxcabazepine; *PGB* Pregabalin; *PHE* Phenytoin; *RUF* Rufinamide; *TPM* Topiramate

In the five seizure-free patients, CNB was added to the combinations of 1) lacosamide and brivaracetam, 2) lacosamide and clobazam, 3) levetiracetam and lamotrigine, 4) levetiracetam, zonisamide and lacosamide, and 5) lamotrigine, levetiracetam and lacosamide. Three out of five seizure-free patients had CNB added as the third drug, while the last two had CNB introduced as the fourth drug.

The ASMs most commonly combined with CNB in the group of responders with a 75–99% seizure reduction were lacosamide (3/3) and zonisamide (2/3). CNB was added as the third drug in 2/3 patients and the fifth drug in 1/3 patients.

In the group with a 50–74% reduction in seizure frequency (6/14 responders; 18,8% of the total cohort) CNB was most often combined with lacosamide (4/6) or levetiracetam (3/6) and introduced as the third drug in 1/6 patients, as the fourth drug in 2/6 patients, and as the fifth drug in 2/6 patients, while the last patient had CNB added as the sixth drug.

CNB was most often combined with lamotrigine (3/7), lacosamide (4/7), or levetiracetam (3/7) in the group of non-responders of which 4/7 patients had CNB added as the third drug, 2/7 had CNB added as the fourth drug, and 1/7 had CNB added as the fifth drug.

Of the 11 patients who discontinued CNB, the most frequently concomitant ASMs were levetiracetam (7/11), lacosamide (3/11), and topiramate (3/11). CNB was most often introduced as the third (4/11) and the fifth drug (3/11). Of the 31 adjustments of concomitant ASMs in total, only five were performed in the group of patients who discontinued CNB.

### Safety and tolerability

Overall, 15/32 (47%) patients reported at least one adverse effect during the treatment period, and the most reported were tiredness (13/15) and dizziness (9/15). In 5/15 (33%) cases, the adverse effects were resolved by a reduction in either concomitant ASMs or by a reduction in CNB dose. In the remaining 10/15 (67%) cases, CNB was discontinued due to unresolved adverse effects (psychiatric symptoms, diarrhea, tiredness) including three patients who also did not respond to the treatment. An adverse effect was reported by 10/15 (67%) patients at doses of CNB of 25–200 mg; and it led to CNB discontinuation in nine of them, on patients’ request. The lowest percentage of patients reporting at least one adverse effect during the treatment period was found in the group of patients with CNB added as the third drug (Table 3). No patients developed severe adverse events or DRESS.

**Table 3:** Summary of adverse effects in the total cohort of 32 patients in comparison with different maintenance doses of cenobamate

Summary of adverse effects	Total cohort (*n* = 32)	Patients with discontinued treatment (*n* = 11)
≥ 1 reported adverse effect at any time during the treatment period	16 (47%)	11 (100%)
• Tiredness, n (%)	13 (41%)	6 (55%)
• Dizziness/impaired balance, n (%)	9 (28%)	5 (45%)
• Psychiatric symptoms, n (%)	2 (6%)	1 (9%)
• Diarrhea, n (%)	1 (3%)	1 (9%)
• Increased seiure frequency, n (%)	0 (0%)	1 (9%)
CNB dose when first reported adverse effect		
• 25 mg	2 (6%)	2 (18%)
• 50 mg	2 (6%)	2 (18%)
• 100 mg	4 (13%)	4 (36%)
• 200 mg	2 (6%)	3 (27%)
• 250 mg	1 (3%)	0 (0%)
• 300 mg	1 (3%)	0 (0%)
• 350 mg	2 (6%)	0 (0%)
• 400 mg	1 (3%)	0 (0%)
≥ 1 reported adverse effect reported after CNB was added as		
• Second drug (of the total cohort 2/32)	2/2 (100%)	2/11 (18%)
• Third drug (of the total cohort 14/32)	5/14 (36%)	3/11 (27%)
• Fourth drug (of the total cohort 7/32)	4/7 (57%)	2/11 (18%)
• Fifth drug (of the total cohort 7/32)	4/7 (57%)	3/11 (27%)
• Sixth drug (of the total cohort 2/32)	1/2 (50%)	1/11 (9%)

## Discussion

Across the performed RCTs, CNB was found to reduce seizure frequency by at least 50% in 50.1% of the patients randomized to treatment, and a rate of seizure freedom of about 20% was observed [12]. In open-label extension studies with CNB [10] 11, the median exposure (including the duration of phase 3 studies) was 30.2 and 53.9 months. Among the observed patients in those studies, 13.1% and 16.4% achieved a 100% seizure reduction. Real-world studies reporting the use of CNB in heterogeneous populations of patients with drug-refractory focal epilepsies have been published previously, contributing to expanding the knowledge on the use of CNB in daily practice. These studies [18–26] report a maximum exposure to CNB of 21 months. At variance with most of the published studies, our retrospective, real-world study focused on a particularly severe group of patients suffering from ultra-refractory focal epilepsy, assessing the effectiveness and tolerability of CNB as an add-on treatment for up to 32 months after CNB initiation, and with a mean exposure to CNB of 21 months, which is the longest reported so far in a real-world study.

### Efficacy and tolerability of cenobamate in real-world studies

In our selected cohort of patients with ultra-refractory epilepsy, 44% were responders (≥ 50% seizure reduction), including five who achieved seizure freedom. In our study, a ≥ 50% reduction of both FSIA and FSWIA was observed, which suggests that CNB is effective in treating focal seizures of different types. Additionally, adjunctive treatment with CNB reduced the number of patients with active FBTCS in our cohort by 50%, a seizure type that is known to be associated with an increased risk of sudden death in epilepsy (SUDEP).

Among the published real-world reports investigating add-on CNB, three studies [5, 26, 27] investigated patients suffering from ultra-refractory or severe drug-resistant focal epilepsy treated with a mean/median CNB dose of 208–261.9 mg and median number of ASMs at three or more.

Pena-Ceballos et al. [5] included 57 patients with a retention rate of 50/57 (87.7%). Of the 50 patients analyzed in the study, 5.3% became seizure-free, 42.1% experienced a 75–99% reduction, and 28.1% a reduction of 50–74%. A decrease in seizure frequency below 50% was observed in 24.5% of the patients. Of the total cohort, the median dose of CNB was 250 mg/day and the median duration of treatment was 11 months.

Villanueva et al. [27] included 170 patients of which 124 patients were lost to follow-up. The retention rate after 1 year of treatment was 87% of which 13.3% became seizure-free, 17.6% experienced a reduction in seizures at 75–99%, and 17.5% had a reduction of 50–74%. A decrease in seizure frequency below 50% was observed in 37% of the patients. The median dose of CNB was 250 mg/day and the mean duration of treatment was 6.4 months.

An additional real-world study by Steinhoff et al. [26] included 172 patients of which 22 were lost to follow-up. The retention rate after one year of treatment was 80% of which 14% achieved seizure freedom, and 61% a seizure reduction at ≥ 50%. The mean daily dose of CNB was 208 mg and the patients were followed for 12 months. Compared to our cohort of 32 patients, a retention rate of 78% after one year of treatment was reached, and at the end of the study period the retention rate was 66%. In our study, a median CNB dose of 250 mg led to seizure freedom in 15.6% of patients, while 9% experienced a 75–99% reduction of seizures, and an additional 18.8% experienced a 50–74% seizure reduction. A reduction in seizure frequency of less than 50% was observed in 22% of our cohort, which resulted in a responder rate of 43.75% with a mean length of treatment duration of 21 months. The seizure-free rate (15,6%) reported in our study was superior to that reported in other studies investigating patients suffering from ultra-refractory epilepsies [5, 26, 27], although in our cohort we observed a lower retention rate as compared to these reports possibly depending on the longer follow-up of our study. However, a proportion of the included patients were lost to follow-up in the studies by Villanueva et al. and Steinhoff et al. These patients were not included in the analysis; however, it can be speculated that lack of efficacy and/or tolerability issues might be reasons for the missing follow-up. In our study, we included also five patients who had been treated for less than three months, to analyze reasons for discontinuations. All five patients in our study treated less than three months discontinued treatment due to adverse effects. Reports regarding adverse effects in our cohort correspond to the ones reported from previous real-world studies and RCTs (i.e. somnolence, dizziness, behavioral changes, diarrhea), and they were often reported during the first three months of treatment, underlining the importance of slow titration.

The three studies mentioned [5, 26, 27] reached a mean/median CNB dose of 208–261.9 mg/day. In randomized trials, the greatest effects were observed at 400 mg daily. In our study, only six patients reached 400 mg/day. It is therefore possible, that some of our patients might have experienced a greater seizure reduction, and thus greater responder rate, if the dose of CNB had been increased further. Doses of CNB above 400 mg/day have been reported in other reports [26]. However, high doses of CNB are also associated with a higher risk of adverse effects [7]. On the other hand, a significant reduction of seizure frequency has been observed in patients with severe DRE treated with a median dose of CNB of 100 mg/day [20]. This phenomenon was also present in our cohort, as one patient became seizure-free with add-on CNB of 100 mg. The remarkable response to a low dose of CNB in these patients may be due to a particular sensitivity to the novel GABAergic mode of action of CNB [26].

### Impact on concomitant ASMs and pharmacological challenges with cenobamate

In the study by Pena-Ceballos, the most commonly prescribed ASMs at baseline were clobazam (59.6%), eslicarbazepine (47.4%), lamotrigine (43.9%), and brivaracetam (28.1%), while the most commonly prescribed ASMs in the study by Villanueva et al. were lacosamide (35.9%), brivaracetam, (35.3%), carbamazepine (34.1%), and clobazam (31.8%). In our cohort, the most frequent ASMs at baseline were lacosamide (53%), levetiracetam (47%), lamotrigine (28%), and clobazam (28%), thus CNB was more frequently combined with lacosamide and levetiracetam, than with clobazam and lamotrigine. CNB acts as a combined enzyme inducer and inhibitor, which may alter the serum concentrations of concomitant AMSs considerably and unpredictably [28]. Due to those drug-drug interactions, proactive dose adjustments of clobazam, lacosamide, phenytoin (PHT), and phenobarbital (PB) have been recommended when CNB is added [12]. This could indicate that CNB is more tolerable when combined with ASMs other than sodium channel blockers and clobazam. However, our study did not allow us to draw any conclusions about the most tolerable ASM combinations, probably due to the limited size of our cohort. During the study period of 32 months, 19/32 (59%) of the patients in our cohort had concomitant ASMs either changed, reduced in dose, or discontinued. Most of the dose adjustments were carried out to reduce adverse effects or to decrease the drug load after a satisfying seizure reduction was obtained. We may speculate that in some patients a more consistent reduction of concomitant ASMs earlier during the titration of CNB might have increased the tolerability, thus reducing the number of patients who discontinued CNB treatment. In Europe, CNB is only licensed for monotherapy. Given the drug-drug interactions discussed and the high efficacy, CNB as monotherapy or as early add-on might be a promising treatment option.

### Limitations

Some study limitations are worth mentioning. Due to the retrospective design of our study, the treatment with CNB was neither randomized nor blinded. In addition, our study included a relatively small sample of patients, as we aimed only to include patients with ultra-refractory focal epilepsy. In our study, patients and caregivers did not report seizure frequency in a standardized manner, which introduced a degree of heterogeneity in the seizure frequency evaluation, possibly underestimating the actual seizure frequency in some patients. Third, serum concentration measurements were not yet available for cenobamate, thus pharmacokinetic variability could not be controlled.

## Conclusions

Our findings in patients suffering from ultra-refractory focal epilepsies with the longest exposure to CNB to date in a real-world study suggest the usefulness of CNB as an add-on treatment in this very severe group of epilepsies. In our cohort, 15.6% of the patients became seizure-free after add-on treatment with CNB, and 50% of patients suffering from FBTCS at baseline achieved a 100% reduction of this seizure type. At the end of the study period, seizure-free patients were without seizures for a mean period of 15.4 months, which indicates that the efficacy of CNB is long-lasting also in real-world practice. Our study contributes to additional knowledge on the long-lasting effectiveness and tolerability of adjunctive CNB in patients with ultra-refractory focal epilepsies in daily clinical practice. Further studies in patients with epilepsy treated at an earlier stage of their illness are warranted to explore the real impact of CNB and its position in the antiseizure medication armamentarium.

## Data Availability

The datasets generated during and/or analysed during the current study are available from the corresponding author on reasonable request.
